# Underground Signals: The Ecological Power of Root Volatiles

**DOI:** 10.1002/advs.202522148

**Published:** 2026-09-21

**Authors:** Xueyao Cao, Nanli Song, François Verheggen, Meritxell Pérez‐Hedo, Andrea Clavijo McCormick, Antonino Cusumano, Arthur de Fouchier, Bing Wang, Chen‐Zhu Wang, Emmanuelle Jacquin‐Joly, Gary W. Felton, Geoff M. Gurr, Hao Guo, Jiancai Li, Jin Zhang, Li Chen, Manqun Wang, Michele Ricupero, Peng Han, Peng He, Qi Yan, Qingbo Tang, Qingsong Liu, Shiheng An, Xiangbo Kong, Xiaoling Sun, Xueming Ren, Yuanyuan Song, Yang Liu, Yanhui Guo, Yunhe Li, Xiao Sun

**Affiliations:** ^1^ State Key Laboratory of Crop Stress Adaptation and Improvement School of Life Sciences Henan University Kaifeng China; ^2^ Laboratory of Chemical and Behavioral Ecology Gembloux Agro‐Bio Tech University of Liege Gembloux Belgium; ^3^ Instituto de Biología Molecular y Celular de Plantas (IBMCP) Consejo Superior de Investigaciones Científicas Universitat Politècnica de València Valencia Spain; ^4^ School of Agriculture and Environment Massey University Palmerston North New Zealand; ^5^ Department of Agricultural, Food and Forest Sciences (SAAF) University of Palermo Viale delle Scienze Palermo Italy; ^6^ Institute of Ecology and Environmental Sciences of Paris Sorbonne Université INRAE CNRS IRD UPEC Université Paris Cité Versailles France; ^7^ State Key Laboratory for Biology of Plant Diseases and Insect Pests Institute of Plant Protection Chinese Academy of Agricultural Sciences Beijing China; ^8^ School of Synthetic Biology School of Life Science Shanxi University Taiyuan China; ^9^ State Key Laboratory of Animal Biodiversity Conservation and Integrated Pest Management Institute of Zoology Chinese Academy of Sciences Beijing China; ^10^ Department of Entomology The Pennsylvania State University University Park Pennsylvania USA; ^11^ State Key Laboratory for Ecological Pest Control of Fujian and Taiwan Crops Institute of Applied Ecology Fujian Agriculture and Forestry University Fuzhou China; ^12^ Joint International Research Laboratory of Ecological Pest Control Ministry of Education Fuzhou China; ^13^ Gulbali Institute Charles Sturt University Orange New South Wales Australia; ^14^ College of Life Sciences/Hebei Basic Science Center for Biotic Interactions Hebei University Baoding China; ^15^ State Key Laboratory of Plant Trait Design CAS Center for Excellence in Molecular Plant Sciences Shanghai Institute of Plant Physiology and Ecology Chinese Academy of Sciences Shanghai China; ^16^ State Key Laboratory of Agricultural and Forestry Biosecurity College of Plant Protection Nanjing Agricultural University Nanjing China; ^17^ Key Laboratory of Integrated Management of Crop Disease and Pests (Ministry of Education) Nanjing Agricultural University Nanjing China; ^18^ Key Laboratory of Soybean Disease and Pest Control (Ministry of Agriculture and Rural Affairs) Nanjing Agricultural University Nanjing China; ^19^ Hubei Insect Resources Utilization and Sustainable Pest Management Key Laboratory College of Plant Science and Technology Huazhong Agricultural University Wuhan China; ^20^ Department of Agriculture Food and Environment University of Catania Catania Italy; ^21^ Yunnan Key Laboratory of Plant Reproductive Adaptation and Evolutionary Ecology Laboratory of Ecology and Evolutionary Biology School of Ecology and Environmental Sciences Yunnan University Kunming China; ^22^ State Key Laboratory of Green Pesticide Center For R&D of Fine Chemicals of Guizhou University Guiyang China; ^23^ Department of Entomology College of Plant Protection Henan Agricultural University Zhengzhou China; ^24^ Laboratory of Cotton Bio‐Breeding and Integrated Utilization School of Life Sciences College of Agriculture Henan University Kaifeng China; ^25^ Henan International Laboratory for Green Pest Control College of Plant Protection Henan Agricultural University Zhengzhou China; ^26^ Key Laboratory of National Forestry and Grassland Administration on Forest Ecosystem Protection and Restoration Ecology and Nature Conservation Institute Chinese Academy of Forestry Beijing China; ^27^ Tea Research Institute，Chinese Academy of Agricultural Sciences State Key Laboratory of Tea Plant Germplasm Innovation and Resource Utilization Hangzhou China; ^28^ Key Laboratory of Ministry of Education for Genetics Breeding and Multiple Utilization of Crops College of Agriculture Fujian Agriculture and Forestry University Fuzhou China; ^29^ Key Laboratory of Biological Breeding for Fujian and Taiwan Crops Ministry of Agriculture and Rural Affairs Fujian Agriculture and Forestry University Fuzhou China

**Keywords:** belowground chemical networks, multitrophic interactions, plant‐microbe‐invertebrate communications, rhizosphere ecology, root volatiles, sustainable agriculture

## Abstract

Root volatiles mediate diverse belowground interactions among plants, microbes, and invertebrates, but biological and environmental conditions strongly influence their production, movement, perception, and ecological effects. This review synthesizes current knowledge through an integrative conceptual framework linking five dimensions: (1) chemical diversity and biosynthesis; (2) regulation by biotic and abiotic factors; (3) plant internal signaling and resource allocation; (4) ecological adaptation and reciprocal evolutionary interactions with soil organisms; and (5) implications for agricultural management. Key roles include defense against herbivores and pathogens, recruitment of beneficial microbes and natural enemies, plant recognition, allelopathy, systemic signaling between roots and shoots, and coordination across multitrophic networks. The synthesis examines how volatile identity and concentration, soil properties, microbial transformation, and plant genotype influence signal transmission and ecological outcomes. Comparisons across taxa, compounds, and experimental systems clarify which mechanisms are broadly supported and which remain context‐dependent. Priorities include resolving molecular pathways, tracing volatile transport and persistence in soil, distinguishing volatile effects from those of nonvolatile exudates, and validating functions under field conditions. A deeper mechanistic and ecological understanding of root volatiles is therefore essential for developing resilient crop protection strategies, reducing dependence on agrochemical inputs, advancing sustainable agroecology, and sustaining long‐term soil health.

## Graphical abstract

Root volatiles mediate rhizosphere chemical communication, shaping interactions among plants, microbes, herbivores, and natural enemies. Their production, transport, and perception underpin ecological adaptation, multitrophic interactions, and plant resilience, offering opportunities for sustainable crop protection, resilient agroecosystems, and improved soil health.

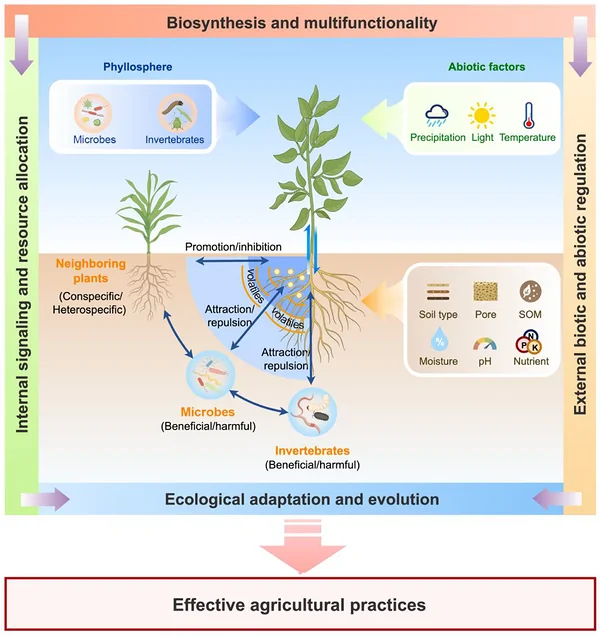

**Abbreviations**
12‐OPDA: 12‐oxo‐phytodienoic acid
ACC: 1‐aminocyclopropane‐1‐carboxylic acid
AAT: alcohol acyltransferase
ADH: alcohol dehydrogenase
ALDH: aldehyde dehydrogenase
DMAPP: dimethylallyl diphosphate
EO: essential oil
EPN: entomopathogenic nematode
FPP: (*E,E*)‐farnesyl diphosphate
GC‐MS: gas chromatography‐mass spectrometry
GSL: glucosinolate
IPP: isopentenyl diphosphate
LOX: lipoxygenase
MeJA: methyl jasmonate
MEP: methylerythritol phosphate
MeSA: methyl salicylate
MVA: mevalonate
PTR‐MS: proton transfer reaction‐mass spectrometry
SOM: soil organic matter
TPS: terpene synthase

## 1 Introduction

Plants release up to 10% of their fixed carbon into the atmosphere as phytogenic volatiles [1, 2, 3]. Plant volatiles serve critical ecological roles in plant defense, chemical communication, and species competition [4, 5, 6, 7, 8, 9, 10]. Synthesized across diverse plant tissues via various biochemical pathways, plant volatiles exhibit remarkable chemical diversity, encompassing over 30 000 distinct substances, including alkanes, alkenes, alcohols, aldehydes, ethers, esters, and carboxylic acids [2]. Although research on phytogenic volatiles has been dominated by studies of foliar volatiles, this review focuses on root‐derived volatiles, which, despite being less investigated, play key roles in structuring underground chemical networks.

Advances in chemical ecology, microbiology, and invasion biology have established root volatiles as crucial regulators of interactions among conspecific and heterospecific plants, soil microbes, and invertebrate communities [11, 12, 13, 14, 15, 16, 17, 18]. Roots emit a diverse array of volatile compounds, ranging from simple inorganic gases like carbon dioxide (CO_2_) to complex organic molecules such as terpenoids and phenolics [12, 14, 19, 20, 21]. More recently, mounting evidence suggests that root volatiles are not mere metabolic byproducts but functionally significant signals. Similar to aboveground volatiles, root volatiles likely exhibit high levels of signal specificity, even at extremely low compound concentrations [22, 23, 24]. There is increasing advocacy for a deeper investigation of root volatiles due to their profound ecological and agricultural implications [25, 26, 27, 28]. Aboveground volatiles orchestrate intricate aerial networks, modulating vertebrate and invertebrate behavior and priming defenses in neighboring plants–topics that have been extensively reviewed [29, 30, 31, 32, 33, 34, 35]. Root volatiles similarly mediate plant‐invertebrate and plant‐microbe interactions [17, 21, 36, 37, 38, 39], while driving plant–plant communication and abiotic stress responses [13, 16, 40]. Empirical studies confirm that root volatiles are multifunctional regulators of plant adaptation, emitted constitutively or induced by stressors such as herbivory or nutrient scarcity. To cope with biotic stress, root volatiles can act either directly by deterring herbivores, inhibiting pathogens, and suppressing competitors [41, 42, 43], or indirectly by recruiting natural enemies of herbivores and beneficial microbes [24, 37]. In this context, biotic stresses induce temporal dynamics in volatile emissions and derive from both belowground and aboveground sources, suggesting the potential of root volatiles to mediate shoot–root signaling [44, 45, 46, 47]. Under abiotic stress, root volatiles often operate indirectly, primarily by shaping microbial communities to enhance nutrient acquisition and stress tolerance [16, 48]. Notably, producing root volatiles is not without costs. Investment in root volatile production may occur at the expense of plant growth [49]. Indeed, reducing volatile emissions under nutrient limitation, thereby conserving resources and maintaining growth, is an adaptive strategy in plants [50].

Despite increasing recognition of their ecological role and practical significance, the study of root volatiles continues to face substantial technical and conceptual challenges. Unlike foliar volatiles, root volatiles are released into a complex, heterogeneous soil matrix [51], making their collection, quantification, and identification more arduous. Soil components can adsorb volatiles, while soil physicochemical properties and microbial activity can modify or degrade them, contributing to detection issues [19, 21]. Furthermore, their typically low, transient, and context‐dependent concentrations demand highly sensitive and standardized sampling methods [52, 53]. Beyond these technical hurdles, the tendency to study pairwise interactions in isolation has constrained our understanding of the complex ecological networks mediated by root volatiles. These limitations have historically impeded the establishment of a unified framework for both our understanding and application of belowground volatile signaling.

**FIGURE 1:**
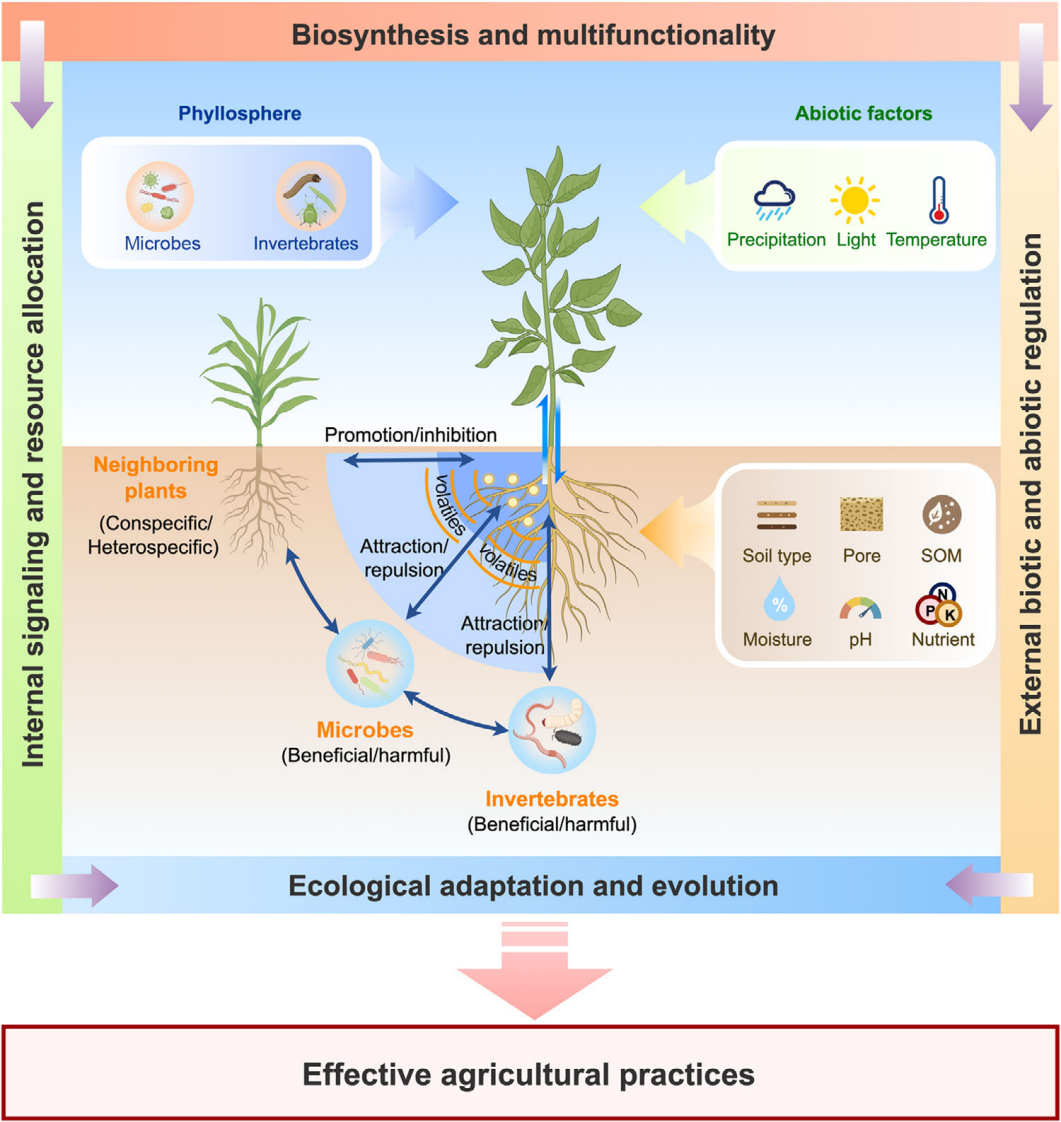
Conceptual framework linking the formation, environmental regulation, ecological functions, evolutionary implications, and agricultural applications of root volatiles. The framework illustrates how root volatiles mediate interactions among plants, invertebrates, and microbes under changing biotic and abiotic conditions. It comprises five interconnected dimensions: (1) the chemical diversity, biosynthesis, release, and ecological functions of root volatiles; (2) the regulation of root volatile production, transport, persistence, and activity by biotic and abiotic environmental factors; (3) internal plant responses associated with root volatile signaling, including systemic signaling and resource allocation trade‐offs; (4) ecological adaptation and reciprocal evolutionary interactions between plants and soil organisms; and (5) the translation of this knowledge into biological control, crop improvement, and soil management. Together, these dimensions connect the mechanistic basis of root volatile signaling with its ecological, evolutionary, and agricultural consequences. By Figdraw.

This review examines root volatiles as mediators of belowground interactions under diverse environmental pressures, integrating mechanistic and ecological insights to highlight their multifunctionality in rhizosphere dynamics. Beyond the mechanistic understanding of root volatile function, it is equally important to consider their origin and role in long‐term ecological dynamics. Therefore, from broader ecological and evolutionary perspectives, we propose a conceptual framework to understand root volatile‐mediated belowground interaction networks. This framework integrates five interconnected aspects: (1) the biosynthesis and multifunctionality of root volatiles; (2) regulation of root volatiles by external biotic and abiotic environmental factors; (3) root volatile‐associated plant internal signaling and resource allocation trade‐offs; (4) ecological adaptation and evolution between root volatiles and soil biota during long‐term coexistence; and (5) the significance of understanding the above four aspects for effective agricultural practices (Figure 1). Within this framework, this review aims to synthesize current advances, identify key knowledge gaps, and provide directions for future research and application of root volatiles. Ultimately, these insights may provide informed guidance on sustainable strategies for soil environment management, particularly for optimizing agricultural practices.

## 2 Properties and Constraints of Root Volatile Signaling

### 2.1 Chemical Diversity and Biosynthesis

**FIGURE 2:**
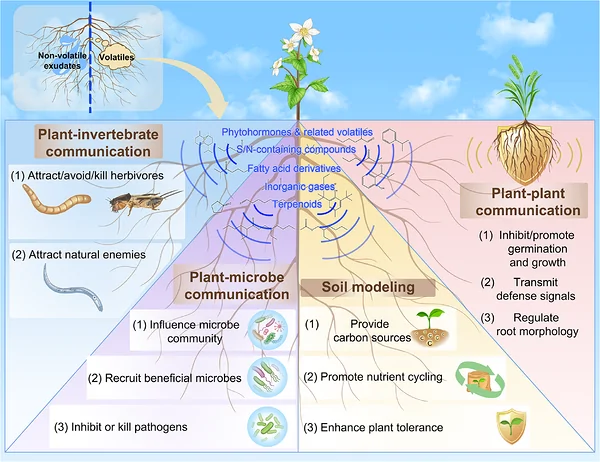
Ecological functions and interaction networks of root volatiles across terrestrial ecosystems. Root‐released chemical compounds are divided into nonvolatile exudates and volatiles, while the interaction pathways shown in the figure focus specifically on signaling mediated by root volatiles. Four principal pathways are illustrated. (1) Interactions between plants and invertebrates: root volatiles can attract or repel root‐feeding herbivores and can recruit natural enemies, including entomopathogenic nematodes and predatory arthropods. (2) Interactions between plants and microbes: root volatiles can influence microbial growth, colonization, and community composition, promote the activity of certain beneficial microbes, and inhibit selected soil pathogens. (3) Effects on soil processes: some root volatiles can be metabolized by soil microbes, contribute to carbon transformation and nutrient cycling, and influence plant responses to abiotic stress. (4) Interactions between plants: root volatiles can inhibit or promote the growth of neighboring plants, influence root system architecture, and transmit information associated with environmental stress or herbivore attack. These functions are mediated by several classes of root volatiles, including terpenoids, fatty acid derivatives, sulfur‐ and nitrogen‐containing compounds, inorganic gases, and volatile phytohormones and related compounds. Together, these pathways connect biological interactions across trophic levels and link aboveground and belowground processes.

Root volatiles exhibit remarkable chemodiversity, encompassing numerous chemical classes derived from diverse biosynthetic pathways [14, 21, 54]. These compounds can be broadly categorized into the following major groups (Figure 2):

1. Terpenoids are mainly synthesized from isopentenyl diphosphate (IPP) and dimethylallyl diphosphate (DMAPP) through the mevalonate (MVA) and methylerythritol phosphate (MEP) pathways. These processes involve the formation of prenyl diphosphate precursors and their conversion by terpene synthases (TPSs) or further modification by cytochrome P450 enzymes [55, 56, 57, 58]. Representative examples include the root‐specific production of the monoterpene 1,8‐cineole in *Arabidopsis thaliana* by a dedicated root‐expressed TPS gene [59], and the formation of the volatile sesquiterpene (*E*)‐β‐caryophyllene in maize roots by the sesquiterpene synthase gene*TPS23* [60].
2. Fatty acid derivatives are generated through the lipoxygenase (LOX) pathway, in which linoleic and α‐linolenic acids are oxidized to hydroperoxy intermediates and subsequently cleaved into aldehydes. These aldehydes are then reduced to alcohols by alcohol dehydrogenases (ADHs), further converted into esters by alcohol acyltransferases (AATs), or oxidized to fatty acids by aldehyde dehydrogenases (ALDHs) [61, 62, 63]. For example, barley roots showed a direct association between LOX activity and the formation of root‐emitted volatile aldehydes [64], while metabolome‐transcriptome analysis of *Bupleurum scorzonerifolium* roots further supported this biosynthetic route from pathway to gene level [63].
3. Sulfur‐ and nitrogen‐containing compounds are mainly derived from amino acid‐based glucosinolate (GSL) metabolism. Methionine‐, tryptophan‐, and phenylalanine/tyrosine‐derived GSLs are stored as nonvolatile precursors and hydrolyzed upon tissue disruption or root herbivory to generate volatiles [65, 66, 67]. Examples include rutabaga and turnip roots, where GSL hydrolysis produced volatile isothiocyanates and nitriles [68], and *Arabidopsis* roots, where nitrile‐specifier proteins promoted GSL breakdown into simple nitrile products [69].
4. Inorganic gaseous compounds mainly originate from primary nitrogen and carbon metabolism. NO can be produced through nitrate reductase‐mediated conversion of nitrate to nitrite, followed by nitrite reduction [70, 71], whereas CO_2_ is continuously generated as the end product of root respiration [72]. Although other gases, such as H_2_S and H_2_, have been reported in plant root tissues [73, 74], their emission from roots into the rhizosphere has not been conclusively demonstrated, and their ecological roles as root‐emitted gases remain largely speculative.
5. Phytohormones and related volatile compounds, including ethylene, methyl salicylate (MeSA), and methyl jasmonate (MeJA), have been detected in emissions from plant roots. Although their biosynthetic pathways in root tissues remain insufficiently characterized, evidence from other plant tissues provides a basis for describing their canonical biosynthetic routes. Ethylene is synthesized from methionine via its direct precursor 1‐aminocyclopropane‐1‐carboxylic acid (ACC), with ACC oxidase catalyzing the final step [75]. MeSA and MeJA are derived from the phenylpropanoid and the 12‐oxo‐phytodienoic acid (12‐OPDA) pathways, respectively [76].

Advances in molecular biology have enabled the identification and functional characterization of genes involved in root volatile biosynthesis. In terpenoid metabolism, TPS gene families and their encoded enzymes have been functionally characterized as major contributors to the diversity of volatile terpenoids. In *Artemisia pallens*, a terpene synthase (ApTPS1) catalyzed the formation of β‐elemene and germacrene A with (*E,E*)‐farnesyl diphosphate (FPP) as a substrate in roots [55]. In addition, herbivory‐induced volatile monoterpene emissions in poplar (*Populus trichocarpa*) roots have been associated with the functionally characterized terpene synthases (PtTPS16 and PtTPS21) [77]. Similarly, an integrated metabolomic and transcriptomic analysis of *B. scorzonerifolium* roots revealed that volatile variation during development was closely associated with differentially expressed genes involved in oxylipin metabolism, suggesting transcriptional regulation of LOX‐related pathways in root volatile biosynthesis [63]. Furthermore, sulfur‐ and nitrogen‐containing volatile precursor pathways, particularly GSL biosynthesis, are transcriptionally regulated under stress conditions. The expression of indole GSL biosynthetic genes, such as *CYP79B2*, has been reported to increase following insect herbivory [66]. These regulatory modules offer promising targets for genome‐editing tools to enhance plant resilience by manipulating root volatile profiles [78, 79].

Overall, current knowledge on the genetic basis of root volatile biosynthesis is unevenly distributed across compound classes. Terpenoid biosynthesis has been relatively well characterized due to its ecological importance and the availability of functionally validated genes in multiple species. In contrast, other classes of root volatiles remain less explored. More studies are therefore needed for a balanced understanding of root volatile metabolism. Integrating this molecular understanding with ecological perspectives could pave the way for developing crop varieties with optimized belowground communication networks.

### 2.2 Diffusion Dynamics and Soil‐Mediated Constraints

Previous reviews have mainly discussed individual factors affecting volatile diffusion in soil [13, 38, 52]. Here, these factors are integrated into two key aspects: (1) the accessible space for root volatile transport and (2) the adsorption and degradation of root volatiles.

(1) The accessible space for root volatile transport is primarily governed by the properties of volatiles and the pore network of the soil. According to the “like‐dissolves‐like” principle, nonpolar compounds exhibit low water solubility and are therefore predominantly transported via the air space, leading to rapid diffusion. In contrast, more polar compounds, owing to their greater water solubility, can diffuse simultaneously through both the air and water [80, 81]. Further, because volatile compounds diffuse much more rapidly through air than water [82], the availability and connectivity of air space in soil become key determinants of root volatile transport. The pore network is an important factor influencing soil air space. Macropores are generally air‐filled and facilitate rapid gas transport, whereas micropores retain water and therefore restrict gaseous diffusion [21]. Consequently, the proportions and spatial distributions of pores with different sizes strongly influence the diffusion efficiency of root volatiles.

Soil moisture content is an important factor influencing soil pore networks. Studies have shown that increased moisture content induces the disintegration of aggregates, resulting in more small particles that occupy larger pores, thereby significantly affecting pore geometry and connectivity [83]. Soil texture further influences the pore network by determining pore‐size distribution and total porosity [84, 85]. Soil biological activity, including microbial processes and soil fauna burrowing, can alter soil structure and pore architecture, such as microbial regulation of aggregate formation through extracellular polymers and hyphal networks [86]. Unlike soil texture, which largely determines the inherent pore network, biological activity may continuously remodel the accessible diffusion space, thereby making volatile transport a potentially dynamic process.

(2) The adsorption and degradation of root volatiles are primarily governed by volatile properties, soil organic matter (SOM), and soil microbial processes. In soil environments, many volatile compounds are bound to SOM. Differences in vapor pressure among these compounds lead to varying release patterns in response to temperature fluctuations, thereby resulting in distinct diffusion dynamics [87]. SOM formation is jointly controlled by organic input chemistry, soil physicochemical conditions, and decomposer activity, including microbial and soil fauna processes [88], which indirectly regulate the sorptive capacity and temporal dynamics of volatiles in soils. Soil microbial degradation or transformation of volatile organic compounds may influence their transport dynamics by attenuating concentration gradients and shortening their effective signaling distance in soil. Radioisotope (^14^C) labeling studies have demonstrated that several biogenic volatile organic compounds can serve as potential carbon substrates for soil microbes and are rapidly mineralized to CO_2_ [89, 90]. Moreover, soil chemical properties such as pH may further modulate the degradation and transformation of volatiles. For example, linalool can be converted to α‐ and β‐pinene under acidic conditions [91].

Considering both the accessible space for transport and adsorption‐degradation processes, root volatile diffusion in soil is proposed to be jointly governed by volatile properties, soil physicochemical characteristics, and biological activity. However, current understanding still relies heavily on indirect evidence, and more studies using root volatile systems are needed to further validate and quantify their diffusion dynamics under realistic soil conditions. Such knowledge would provide a basis for controlling and monitoring the stability and persistence of root volatile‐based products during application.

### 2.3 Reliability and Specificity of Signals in Heterogeneous Soils

Although volatile properties and soil heterogeneity strongly influence diffusion, root volatiles can still support reliable and specific signaling in soil environments [92]. This signaling capacity is reflected in the host specificity of soil organisms and in their responses that depend on concentration and diversity of volatile blends.

CO_2_ produced by root respiration is a ubiquitous cue for root‐feeding organisms. Many soil‐dwelling herbivores exhibit remarkable sensitivity to minute increases in CO_2_ levels above ambient conditions, migrating along gradients toward respiring roots. For example, the larvae of the western corn rootworm (*Diabrotica virgifera virgifera*) oriented toward CO_2_, which serves as an indicator of nearby respiring roots [93]. While CO_2_ provides a general signal of living tissue, it lacks host specificity [94]. Specific root volatiles complement CO_2_ cues, enabling discrimination among plant species. For example, larvae of *Melolontha* species detected complex volatile blends via sensory appendages [95, 96]. Wireworms were strongly attracted to root volatiles, particularly simple aldehydes such as hexanal [20, 97, 98]. Furthermore, among phytohormone‐related volatiles, MeSA from tomato (*Solanum lycopersicum*) roots was more attractive than volatiles released by spinach roots [99].

The behavioral responses of root herbivores to individual volatiles are also concentration‐dependent. *Agriotes sordidus* larvae were attracted to low concentrations of hexanal and (*E*)‐2‐hexenal, but avoided these compounds at higher concentrations, indicating a reverse dose‐dependent effect [97]. This suggests that root volatile profiles help insects assess host quality or identity. Additionally, the complexity of root volatile blends may influence herbivore responses [100], with some species showing stronger attraction to heterogeneous mixtures compared to uniform profiles, potentially indicating host suitability [101].

In general, root‐emitted volatiles provide long‐range foraging cues, effectively guiding herbivores through heterogeneous soil environments to nutrient‐rich roots. However, whether similar volatile‐mediated processes occur in plant‐associated soil pathogens remains unclear. Given that nonvolatile root exudates are known to attract certain soil‐borne pathogens [102], root volatiles may also serve as cues that enable pathogens to locate suitable host roots.

## 3 Functional Roles of Root Volatiles

Belowground chemical communication is shaped by both nonvolatile root exudates and gaseous root volatiles [25, 39, 103, 104]. Owing to their distinct physicochemical properties, these root‐derived chemical cues differ substantially in soil residence time, mobility, and diffusion range [103], which may determine how long and how far they function in soil systems. Despite these differences, root volatiles remain crucial mediators of belowground plant‐organism interactions [19, 21, 25, 81, 105]. Based on their ecological functions, the roles of root volatiles in belowground systems can be broadly categorized into three aspects: (1) defense against herbivores and pathogens, (2) recruitment of beneficial microbes and natural enemies, and (3) plant recognition and allelopathic interactions (Figure 2).

### 3.1 Defense Against Herbivores and Pathogens

Plants are continuously exposed to attacks from both herbivores and pathogens in the soil environment. In this context, root volatiles can serve as important components of direct plant defense.

Against soil herbivores, root volatiles primarily function through repellency and toxicity. For example, volatile isothiocyanates derived from GSLs, which are predominantly found in Brassicaceae species, contribute substantially to belowground defense against soil herbivores [106, 107, 108, 109]. A more recent study has shown that root volatiles from *Asarum sieboldii* exhibit significant lethal activity against the root‐knot nematode *Meloidogyne incognita*, effectively reducing both the number of root galls and egg masses on tomato roots [43]. In addition, it has been proposed that different root volatile compounds may selectively mediate repellency and toxicity. Specific root volatiles from the wild relative *Cucumis metuliferus* exhibited distinct bioactivities: (methoxymethyl)‐benzene acted as a strong repellent, whereas creosol and (*Z*)‐2‐penten‐1‐ol displayed concentration‐dependent nematicidal effects against *M. incognita* [110]. By acting as allomones that directly repel or intoxicate pests, root volatiles can establish a chemical barrier against herbivores and thereby benefit the plant.

Beyond their role in defending against soil herbivores, root volatiles also contribute to plant protection against soil‐borne pathogens. This protective role primarily manifests in the suppression of pathogen growth [111]. A plant‐pathogen coculture assay showed that volatiles released from *Fusarium culmorum*‐infected barley (*Hordeum vulgare*) roots reduced the growth of *Cochliobolus sativus* by 13%–17% [42]. In most cases, however, the antimicrobial potential of root volatiles has been inferred from in vitro assays using synthetic standards of specific compounds. Representative compounds, including octanol, nonanal, and decanal from pineapple (*Ananas comosus*) [112], 1,8‐cineole and (‐)‐β‐pinene from western balsam poplar [77], and benzothiazole and (*E,E*)‐2,4‐heptadienal from *Codonopsis pilosula* [113], exhibited inhibitory effects against multiple soil‐borne pathogens. These findings suggest that root volatiles constitute an important component of belowground chemical defense, acting directly on pathogen growth and thereby potentially contributing to a suppressive rhizosphere microenvironment.

Current understanding of direct belowground defense mediated by root volatiles is still partly derived from functional assays using essential oils (EOs) extracted from roots, or synthetic standards rather than naturally emitted root volatile blends. Indeed, EOs from diverse plant species have been reported to possess insecticidal and antimicrobial activities [114]. However, studies on belowground herbivores have focused mainly on plant‐parasitic nematodes, leaving other soil herbivores relatively underexplored. Although assays using EOs or synthetic compounds are valuable for identifying bioactive metabolites and developing potential applications, they do not fully recapitulate natural volatile emission from living roots [114, 115]. This limitation constrains our understanding of how plants directly defend themselves against belowground biotic stresses under ecologically realistic conditions.

### 3.2 Recruitment of Beneficial Microbes and Natural Enemies

Beyond their direct defensive roles, root volatiles also contribute to indirect defense by recruiting natural enemies and beneficial microbes. This indirect defense strategy closely mirrors aboveground systems, in which herbivore‐induced plant volatiles attract parasitoids or predators to reduce herbivory pressure [116, 117, 118, 119]. Belowground, however, the beneficial organisms recruited include predatory insects, entomopathogenic nematodes (EPNs), and beneficial microbes. Moreover, these beneficial microbes can further promote plant growth [21, 48], highlighting the multifunctional roles of root volatiles.

Numerous studies have confirmed that root herbivory can induce the release of volatiles that attract natural enemies of the herbivore. For example, maize roots infested by western corn rootworm larvae released the sesquiterpene (*E*)‐β‐caryophyllene into the soil. This compound did not repel rootworms but attracted EPNs, leading to higher infection rates in rootworm larvae [60, 120]. Maize varieties lacking root emission of (*E*)‐β‐caryophyllene failed to recruit EPNs and suffered greater damage [121]. Similar strategies occur in other crops. For instance, volatiles released from sugarcane (*Saccharum officinarum*) roots damaged by spittlebug nymphs, *Mahanarva fimbriolata*, attracted the EPNs *Heterorhabditis indica* and *Steinernema carpocapsae* [122]. Likewise, the roots of blueberry (*Vaccinium corymbosum*), when attacked by weevil larvae *Aegorhinus superciliosus*, released volatiles such as MeSA and 1‐nonine that attracted the EPN *Steinernema australe* [123]. Although specific bioactive compounds have been identified in many studies, natural root volatile blends may be more attractive than individual volatile compounds. Predatory beetles (*Aleochara bilineata)* were more strongly attracted to the natural volatile blend from *Brassica napus* roots than to dimethyl disulfide presented alone [124]. This suggests that complex volatile blends released by plants may convey more ecologically relevant information than individual compounds.

A similar recruitment strategy also occurs during pathogen infection, in which plants release root volatiles to attract beneficial microbes that help suppress disease. For example, volatiles from *Carex arenaria* roots infected with *F. culmorum* recruited antifungal *Burkholderia* and *Paenibacillus* species [24]. Notably, this attraction is more pronounced under nutrient‐limited conditions, possibly because some root volatiles, such as isoprene and α‐pinene, can serve as supplementary carbon substrates for particular soil microbes [14, 125, 126, 127, 128]. This may also be attributed to the ability of root volatiles to promote the growth and colonization of beneficial microbes, as shown by tomato root volatiles that significantly promoted pre‐symbiotic sporulation of arbuscular mycorrhizal fungi [129]. However, the recruitment of beneficial microbes is not restricted to pathogen‐infected plants. Evidence showed that volatiles released by healthy tomato roots can also attract beneficial bacteria [130]. Consistently, root volatiles released from *C. pilosula* showed positive correlations with *Bradyrhizobium* and beneficial fungi [113]. Importantly, the recruited microbes not only exhibit antifungal activities but also promote plant growth. For instance, ethylene, a volatile hormone emitted by roots, increased the relative abundance of specific actinobacterial species, thereby altering the entire soil microbial network and enhancing seed production [131]. Similarly, MeJA, a bioactive signal among root volatiles, can rapidly trigger biofilm formation, thereby providing distance‐dependent benefits to the host by promoting plant growth [48]. These interactions establish a dynamic plant‐beneficial microbe‐pathogen network, which enhances plant defense against soil‐borne diseases while simultaneously promoting plant growth.

Collectively, these findings highlight the potential of root volatiles in biological control and plant growth promotion. Studies on natural enemy recruitment have mainly focused on EPNs, whereas belowground predatory arthropods and other non‐nematode natural enemies are underexplored. Evidence for beneficial microbe recruitment is also limited, and how root volatiles selectively recruit beneficial microbes in realistic soil environments and influence plant health remains unclear.

### 3.3 Plant Recognition and Allelopathic Interactions

Root volatiles provide a chemical interface for belowground plant–plant interactions, linking plant recognition, allelopathic regulation, and stress‐related signaling. By acting as diffusible chemical cues, they enable neighboring plants to perceive surrounding individuals and adjust root growth, competitive behavior, and defensive responses [13, 105, 132]. These volatile‐mediated processes highlight plants' capacity to sense and respond to chemical signals in the belowground environment, which contributes to the adaptive organization of root systems.

In root volatile‐mediated plant–plant communication, individuals can be generally divided into emitters and receivers [17, 40, 119]. Competition is a common consequence of coexistence among different plant species. Under such conditions, allelopathy is an important strategy by which emitters suppress neighboring plants, thereby enhancing their own competitive advantage [133, 134]. Root volatiles also play important roles in this process. Volatile terpenes released from the roots of the invasive shrub *Chrysanthemoides monilifera* in New South Wales inhibited seedling growth of the native sedge *Isolepis nodosa* [41]. Similar allelopathic effects have also been observed for root volatiles from *Artemisia tridentata* and *Pinus halepensis* [135, 136]. Importantly, emitter‐derived root volatiles are not confined to allelopathic suppression and may also promote neighboring plant performance. Root‐emitted β‐caryophyllene from *Centaurea stoebe* increased seed germination and biomass accumulation in several neighboring plant species [40]. Interestingly, this positive effect was stronger for heterospecific neighbors than for conspecific plants, suggesting a potential role of root volatiles in species recognition.

From the receiver's perspective, biomass increases triggered by heterospecific root volatiles may represent an active response to neighboring competition. This may occur because plants facing belowground competition tend to allocate more biomass to roots, thereby enhancing their competitive capacity [137]. Consistent with this view, *Taraxacum officinale* showed greater belowground biomass in response to heterospecific than to conspecific root volatiles [17]. This study further revealed that root metabolite profiles were altered by exposure to root volatiles from different neighboring species [17], suggesting that plants can use volatile information to recognize species identity and adjust root metabolism or growth accordingly. Volatile‐mediated interactions may also operate under stress conditions. Receiver plants can sense stress‐associated volatiles released by emitters and pre‐adjust their own physiological or defensive state [25]. Specifically, jasmonic acid‐treated *Picea abies* released enhanced root volatile signals, which then primed early herbivore‐induced defense responses of *Fagus sylvatica* [138]. These findings indicate that root volatiles not only participate in plant identity recognition but also mediate the transfer of stress information among neighboring individuals.

Overall, we synthesize current studies to highlight the important roles of root volatiles in plant–plant communication from both emitter and receiver perspectives. However, it remains unresolved whether neighboring plant responses arise from active emitter regulation, receiver perception and adaptation, or reciprocal feedback between emitters and receivers.

## 4 Multitrophic and Systemic Integration

### 4.1 Plant‐Microbe‐Invertebrate Interaction Networks

Plants, invertebrates, and microbes coexist and form a highly inseparable network of interactions in the soil environment. Accordingly, root volatiles are more likely to function as cross‐kingdom chemical signals that link multiple trophic levels, thereby contributing to the organization of belowground ecological networks.

Root volatiles have been increasingly recognized as multifunctional chemical signals capable of influencing diverse soil biota [14, 19, 105]. Consequently, once released into soil, they are likely to trigger responses across a broad range of organisms. For instance, (*E*)‐β‐caryophyllene from *C. stoebe* increased the growth of the root herbivore *Melolontha melolontha* on *T. officinale*, potentially by modulating root protein and carbohydrate levels in the host plant [17]. When the direct toxicity of root volatiles to herbivores is dominant, contrasting ecological outcomes may emerge. Volatiles from *A. sieboldii* roots reduced the number of *M. incognita* egg masses in tomato [43]. These findings suggest that the observed ecological outcomes cannot be explained by single species responses alone; rather, they likely arise from the combined effects of multiple interacting biotic groups and their cascading responses. Similarly, studies on soil microbial systems further support this complexity. For example, root volatiles have been reported to reshape rhizosphere microbial communities associated with other plants [112]. Moreover, even well‐characterized mutualistic interactions, such as the rhizobia‐bean symbiosis, extend beyond nutrient acquisition, with this symbiosis increasing root herbivore attraction and growth via volatile signals [139].

Despite growing evidence derived from studies of relatively simple biotic interactions, the complexity of root volatile‐mediated belowground ecological networks in multi‐organism communities has been largely overlooked. In particular, how these networks are dynamically structured and how their components interact are unresolved due to a lack of empirical evidence.

### 4.2 Systemic Root–Shoot Signaling

Plants serve as a central hub linking aboveground and belowground ecosystems. In response to changing abiotic conditions and biotic attacks, plants activate systemic responses that are transmitted between the root and shoot compartments [38, 140, 141]. Such systemic root–shoot signaling mediates ecological crosstalk among aboveground and belowground organisms, resulting in synergistic, antagonistic, or integrated outcomes.

Herbivore‐induced root volatiles can mediate systemic defense signaling, bridging belowground and aboveground responses [45, 142, 143]. Root damage by insects has sometimes been shown to trigger changes in volatile emission from undamaged roots or even shoots of the same plant [60, 144, 145]. For example, feeding by western corn rootworm induced stronger production of (*E*)‐β‐caryophyllene from the roots above the feeding site and in adjacent roots, which could help attract EPNs [60]. Similarly, belowground herbivory by *Acalymma vittatum* larvae induced systemic emission of (*E*)‐β‐ocimene from *Cucurbita pepo* shoots, which enhanced neighboring plant resistance against aboveground herbivores [146]. Such systemic defense signals can also be transmitted from aboveground to belowground tissues. Following aboveground feeding by *Spodoptera littoralis*, maize roots were shown to repel western corn rootworm larvae [147].

This cross‐compartment signaling indicates an integrated whole‐plant defense strategy. In addition, root volatiles induced by aboveground herbivory can influence host selection and development of belowground herbivores. *Liriomyza trifolii* attack accelerated the development of soil‐dwelling conspecific pupae via changes in root volatiles [148]. Beyond single‐compartment herbivory, simultaneous attack across above‐ and belowground compartments can amplify plant responses. Consistently, wireworms (*Agriotes* spp.) showed the strongest attraction to roots under dual herbivory involving root feeding by wireworms and foliar attack by *Ostrinia nubilalis* larvae [46].

Current studies on systemic root–shoot signaling mediated by root volatiles have largely focused on herbivore‐induced responses. Nevertheless, in natural aboveground–belowground ecosystems, plants simultaneously interact with diverse organisms, experiencing multiple interacting stresses and signals. For instance, aboveground endophytes can also influence root volatile emission and soil insect behavior [47]. Therefore, understanding how root volatiles contribute to systemic communication under such complex ecological contexts remains a critical challenge, particularly regarding their roles in signal integration and selective information transmission. Given the greater accessibility of the aboveground environment, this knowledge may facilitate aboveground manipulation of root volatiles for pest control and growth promotion.

### 4.3 Context Dependency and Trade‐Offs

Plants must carefully allocate their limited resources among different fitness‐related traits, as investment in one beneficial trait often comes at the expense of another [109, 149, 150]. Root volatiles, which contribute to belowground defense and plant‐organism communication, can also be costly to produce [128]. Therefore, plants face trade‐offs in resource allocation between root volatile emissions and plant growth, as well as among the production of different root volatiles.

Environmental conditions shape resource allocation trade‐offs associated with root volatiles. A notable example comes from Chinese fir (*Cunninghamia lanceolata*), which reduced the release of root volatiles under low‐phosphorus stress to maintain seedling growth [50]. This suggests that, under nutrient limitation, plants can allocate more resources to growth while reducing investment in chemical signal emission. Moreover, as components of plant defense, the costs associated with root volatiles are also consistent with the classical growth‐defense trade‐off theory [151, 152, 153]. For instance, constitutive expression of a terpene synthase gene in maize, leading to continuous emission of (*E*)‐β‐caryophyllene and α‐humulene, impaired plant growth and yield [49]. Such constitutive root volatiles, termed constitutive defenses, generally incur higher costs than inducible defenses activated only upon herbivore attack, and trade‐offs exist between these two defense strategies [154, 155]. Evidence for this trade‐off was observed in *Asclepias syriaca*, in which constitutive root volatile levels were negatively correlated with their inducibility following herbivore attack [156].

The emission of root volatiles is highly context‐dependent, shaped not only by resource availability and stress conditions but also by how plants balance investment in different traits. The costs and benefits of resource allocation related to root volatiles remain poorly understood. Whether such trade‐offs are widespread in natural ecosystems and how they vary under multiple interacting environmental conditions remain important questions for future research. Addressing these questions could help minimize detrimental effects on other key plant performance traits when manipulating root volatile emissions.

## 5 Evolutionary and Ecological Implications

### 5.1 Selection Pressures Shaping Root Volatile Emission

As discussed above, root volatiles play pivotal roles in belowground defense, chemical communication, and multitrophic interactions [19, 21, 25, 81, 105]. However, their production, emission, and ecological functions are not static but are continuously shaped by diverse selective pressures (Figure 1). These pressures arise from biotic and abiotic factors acting across both aboveground and belowground environments.

The external biotic drivers shaping the synthesis and release of root volatiles can be classified into three main categories: microbes, invertebrates, and neighboring plants. (1) Microbes. Typically, colonization of the rhizosphere by symbiotic bacteria and fungi can alter root morphology and volatile emissions [157, 158, 159], whereas pathogen infection (e.g., *F. culmorum*) can induce the release of defensive root volatiles to mitigate damage [24]. More recently, a study revealed that viral infection can also alter root volatile profiles, as observed in tobacco rattle virus‐infected *Nicotiana benthamiana* [160]. (2) Invertebrates. Herbivory by both aboveground and belowground invertebrates can stimulate roots to emit volatiles that either deter or harm herbivores, or attract natural enemies [46, 110, 120]. Moreover, such responses are species‐specific: wild tomato *Solanum pimpinellifolium* released dimethyl disulfide, MeSA, and benzyl alcohol, whereas domesticated tomato *S. lycopersicum* emitted 2‐nonenal and benzyl alcohol following attack by *Spodoptera exigua* [161]. (3) Neighboring plants. Plant coexistence can influence volatile emissions among neighboring individuals [162]. Terpene emissions from leaves of *Rosmarinus officinalis* were lower when the neighbor species was *P. halepensis* [163]. Evidence for neighbor‐induced changes in root volatile emissions, however, remains limited. Among the few reported examples, MeSA has been detected in the shared soil space of mixed *P. abies*‐*F. sylvatica* saplings, whereas no MeSA emission was observed in single‐species cultures [138].

A range of abiotic stresses can also influence root volatile emissions, including drought, waterlogging, salinity, heavy metals, and nutrient limitation. (1) Drought. In pine species, drought can alter the relative abundance of root monoterpenes and sesquiterpenes. These changes may differ among species, with drought increasing monoterpenes but reducing sesquiterpenes in *Pinus strobus* [164], whereas the opposite pattern was observed in *Pinus massoniana* [165]. (2) Waterlogging. Waterlogging creates hypoxic or anoxic conditions in the rhizosphere, profoundly altering root metabolism [166]. Under low‐oxygen conditions, intact roots of tomato, tobacco, and barley exhibited marked increases in NO emission, with rates rising as oxygen availability declined and reaching their highest levels under anoxic conditions [70]. (3) Salinity. The amounts of root volatile aldehydes and monoterpene alcohols in coriander (*Coriandrum sativum*) grown in saline medium increased significantly with increasing NaCl concentrations [167]. Similarly, root isoprene emission in *A. thaliana* also increased in response to increasing NaCl concentrations [168]. (4) Heavy metals. Under MnCl_2_ treatment, *Phytolacca americana* roots released higher amounts of volatiles, and several compounds, including m‐xylene and heptane, were positively correlated with soil Mn concentration. These Mn‐induced root volatiles further reshaped the rhizosphere microbial community, thereby enhancing plant tolerance to Mn stress [16]. (5) Nutrients. The amount of root volatiles released by two Chinese fir families was lower under phosphorus deficiency than under normal supply [50]. This reduction allows plants to allocate more resources to root growth.

In addition to natural environmental drivers, anthropogenic factors such as crop management practices (e.g., cover cropping) and plant domestication have been shown to influence root volatile emissions [169, 170]. These effects are likely mediated, at least in part, through indirect alterations of natural biotic and abiotic conditions. Overall, root volatile emission should be viewed as a dynamic trait that integrates aboveground and belowground environmental cues, thereby coordinating plant interactions with biotic and abiotic surroundings. Nevertheless, current research has focused more on biotic than abiotic drivers, and evidence for abiotic regulation remains limited and fragmented. Several potentially important factors, such as soil pH, have received little attention. A better understanding of how individual and combined environmental drivers shape root volatile emissions will be essential for clarifying their ecological functions in complex natural systems, and for controlling their effectiveness under varying environmental conditions in field applications.

### 5.2 Coevolution With Soil Biota

Root volatiles are not only important mediators of belowground communication but may also reflect evolutionary adaptations arising from interactions between plants and soil biota [128]. Although direct experimental evidence from long‐term studies tracking reciprocal evolutionary changes remains limited, two lines of evidence may provide indications for the occurrence of such coevolutionary processes. First, plants appear to have diversified the composition and emission patterns of root volatiles in association with defense. At the same time, organisms interacting with plant roots exhibit diverse strategies to exploit or manipulate these chemical signals.

As sessile organisms, plants have evolved highly sophisticated adaptive strategies through long‐term natural and artificial selection [171]. Genetic differentiation can therefore arise within species, leading to variation in the emission of root volatiles. Significant differences in the composition or emission rates of root volatiles have been reported among ten accessions of *Achillea collina* [172] and eight tomato cultivars [28]. In crops, artificial selection during domestication or breeding can alter plant defense mediated by root volatiles. Accordingly, cultivated pepper (*Capsicum annuum*) [101] and cultivated tomato [161] exhibited defense‐related root volatiles that differed from those of their wild relatives or natural accessions.

Plants and herbivorous insects represent two of the most diverse groups on Earth, and their extraordinary diversity is hypothesized to have arisen from a long history of reciprocal defense and counter‐defense coevolution [173]. Coexistence histories with herbivores have shaped the chemical diversity of plant root volatiles [174]. In turn, herbivores can exploit these chemical cues through diverse mechanisms to enhance their fitness. Feeding by western corn rootworm provides information that enables conspecific larvae to locate suitable host plants belowground [147]. Larvae can also use root volatiles to assess conspecific density and select hosts with optimal population levels [175]. Notably, pronounced sexual differentiation exists in the ability to perceive root volatiles. Female *Hylastinus obscurus* responded to a broader range of root volatile compounds than males, suggesting that females may be more active during host location through root volatile cues [176].

Similarly, microbes are not merely passive recipients within root volatile‐mediated ecological networks but can actively exploit and even modify plant volatile signals. A recent study showed that *Metarhizium robertsii* can metabolize the root‐derived compound caulilexin C into 1‐methoxyindole, thereby attracting host insects into the rhizosphere [177]. Although caulilexin C is not a volatile compound, this finding suggests that microbes can exploit root‐derived chemical cues. Plant viruses provide a more direct example: tobacco rattle virus infection of *N. benthamiana* induced the production of root volatiles that attracted soil nematode vectors, thereby facilitating viral spread [160].

Together, these studies suggest that root volatiles may participate in evolutionary and reciprocal adaptive processes involving plants and interacting soil organisms. In this context, the ability of soil organisms to exploit root volatiles and the potential mechanisms underlying their evolution represent important yet largely overlooked aspects of belowground interactions. Elucidating these aspects will not only advance our understanding of root volatile‐mediated interaction networks but also provide a theoretical basis for the rational development of root volatile‐based applications while minimizing potential ecological risks.

## 6 Applications and Translational Potential

**FIGURE 3:**
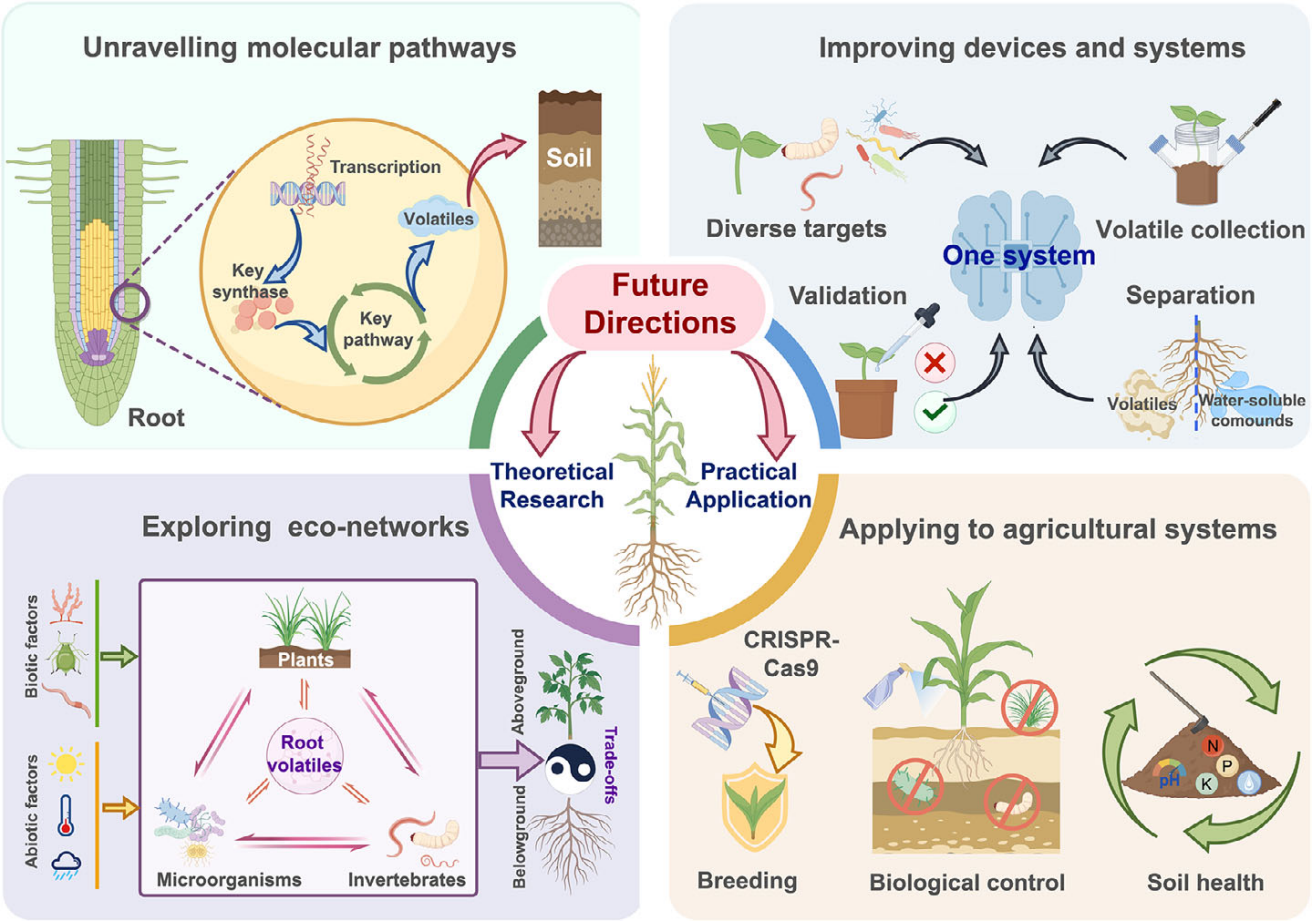
Future research priorities for understanding and applying biological interactions mediated by root volatiles. The framework connects current knowledge gaps with methodological development and agricultural application through four interrelated directions: (1) clarifying the molecular mechanisms underlying root volatile biosynthesis, release, transport, and perception; (2) developing experimental devices and analytical systems that accommodate diverse organisms, enable reliable volatile collection, and support dynamic chemical analysis; (3) moving beyond single‐organism and pairwise studies to investigate interaction networks, internal plant responses, and their underlying mechanisms under multiple biological and environmental conditions; and (4) translating knowledge of root volatiles into agricultural practices through crop breeding, biological control, and soil management. Together, these directions provide a research pathway from mechanistic understanding to ecological validation and practical implementation.

As the molecular mechanisms and ecological functions of root volatiles continue to be unraveled, research is progressively shifting from mechanistic studies toward practical applications. A growing body of research has explored modulating root volatile‐mediated interactions through exogenous application, agricultural management practices, and genetic manipulation. Collectively, these advances underscore the considerable potential of root volatiles for crop protection and sustainable agriculture (Figure 3).

### 6.1 Biocontrol and Agroecological Strategies

Multiple root volatiles with well‐defined biological functions have been identified, providing promising candidates for biological control. For example, (*E*)‐β‐caryophyllene attracts EPNs [60], thymol repels root‐knot nematodes [101], and 1,8‐cineole exhibits antifungal activity [77]. Field studies have further supported the application potential of these compounds. Dimethyl disulfide significantly attracted multiple species of natural enemies under field conditions [124], while 1,5‐dimethylcyclodeca‐1,5,7‐triene increased pest mortality by recruiting four naturally occurring EPN species [26].

Beyond direct application, root volatiles can also be manipulated through agricultural management practices. Cover crops, for example, can alter root volatile emissions [170]. Intercropping with Chinese chive (*Allium tuberosum*) has also been shown to suppress Panama disease (*Fusarium oxysporum* f. sp. *cubense*) through root volatiles [111]. Furthermore, herbivore‐ and pathogen‐induced changes in root volatile emissions hold promise for crop monitoring. For example, *Brassica nigra* exhibited detectable shifts in root volatiles within 1–6 h of *Delia radicum* infestation, suggesting that such rapid responses could provide early indicators of crop damage [44].

Despite these promising advances, the persistence and diffusion of root volatiles are strongly influenced by soil properties, underscoring the need for stable release under diverse soil conditions. Controlled‐release carriers, such as nanomaterials [178], may provide an effective strategy to improve the stability and delivery of root volatile‐based products (e.g., EOs) [179, 180]. For monitoring applications, the performance of root volatile‐based approaches may vary among cultivars [28]. In addition, given the multifunctional nature of root volatiles, product development should consider their potential impacts on nontarget organisms rather than focusing solely on the intended targets.

### 6.2 Breeding and Genetic Manipulation

Several key root volatile biosynthetic pathways have been identified, enabling targeted crop improvement through genetic engineering technologies (e.g., CRISPR/Cas9) [79, 181]. A representative example comes from maize. *TPS23* has been identified as a key gene responsible for the biosynthesis of (*E*)‐β‐caryophyllene, a root volatile compound attractive to EPNs [182]. Researchers successfully restored (*E*)‐β‐caryophyllene production in nonemitting maize varieties by introducing the synthase gene from oregano (*Origanum vulgare*). Field trials further demonstrated that restoring (*E*)‐β‐caryophyllene signaling reduced root damage [27]. However, such genetic manipulation may involve trade‐offs with other plant traits. Subsequent studies showed that introducing this gene could negatively affect seed germination, plant growth, and yield performance [49].

Similar genetic engineering strategies also have the potential to enhance the recruitment of beneficial microbes, improve resistance against pathogens, and promote allelopathic effects against weeds. During this process, the ecological benefits associated with altered root volatile production should be balanced against potential trade‐offs with crop growth, yield, and adaptability to avoid excessive pursuit of a single trait at the expense of overall agronomic performance.

## 7 Outstanding Challenges and Future Directions

Root volatile‐mediated interactions have attracted increasing attention in recent years. However, several fundamental questions remain unresolved. First, the complete pathway linking root volatile biosynthesis, release, soil diffusion, and perception by other organisms is poorly understood. Second, limitations in experimental systems and sampling approaches have constrained the investigation of root volatile‐mediated belowground interaction networks under combinations of biotic and abiotic conditions. These unresolved issues together constrain the practical application of root volatiles. In our opinion, these aspects are interconnected and require systematic, progressive, and balanced investigation. Therefore, we propose a future research framework for advancing root volatile research that encompasses four directions: unravelling molecular pathways, improving devices and systems, exploring ecological networks, and applying to agricultural systems (Figure 3).

### 7.1 Unravelling Molecular Pathways

The biosynthesis, release, and diffusion of root volatiles constitute a continuous biological process, yet each stage is still insufficiently characterized. Recent advances in metabolomics and genomics have revealed the biosynthetic pathways of several important root volatiles [55, 60, 77]. However, root volatiles exhibit remarkable chemical diversity [21, 54], and the biosynthetic pathways of many compounds, as well as their genetic regulation under stress, require further elucidation.

In addition, the transport of volatiles from root tissues to the external environment has received limited attention. Volatile compounds must move from their site of biosynthesis through the plasma membrane and cell wall, and sometimes pass through the cuticle, before being released outside the root [3, 183]. This process likely involves not only concentration‐dependent passive diffusion but also active transport mechanisms. After entering the soil environment, the fate of root volatiles is strongly influenced by soil physical and chemical properties, as well as biological activity [13, 52].

Therefore, to better understand the biosynthesis‐release‐diffusion process of root volatiles, future studies should integrate multi‐omics approaches to investigate the biosynthetic networks and release mechanisms of diverse volatile compounds. In parallel, predictive models incorporating both volatile chemical properties and soil environmental characteristics should be developed to capture their dynamic transport and distribution in soil.

### 7.2 Improving Devices and Systems

Due to the complexity and opacity of belowground environments [51], methodological limitations have long restricted research on root volatiles. Existing experimental systems often lack the capacity to simultaneously support multi‐organism interactions, in situ volatile sampling, and dynamic chemical analysis:

1. Root Volatile Exposure: Current devices are often designed for specific targets and are therefore insufficient for investigating complex underground interaction networks. For instance, sterile two‐compartment Petri dish systems are frequently used to evaluate the effects of root volatiles on specific microbes [42, 48]. Glass olfactometers have been widely applied in studies of soil invertebrates [11, 120] and have recently been adapted for microbial investigations [24, 130].
2. Volatile Collection: Most studies rely on *ex situ* sampling, limiting the ecological relevance of associations between measured volatile blends and their biological functions. Moreover, the detected volatiles are highly dependent on the properties of trapping materials [161]. Common approaches include placing fresh or frozen‐ground root tissues in sealed containers for dynamic headspace sampling or static solid‐phase microextraction (SPME) [20, 47, 110]. Although soil‐embedded SPME has been developed for in situ sampling under both laboratory and field conditions [184, 185], its broader application is limited, possibly due to challenges associated with sample preservation and transportation.
3. Volatile Analysis: Available techniques cannot simultaneously achieve real‐time monitoring and accurate compound identification. Root volatiles are mainly analyzed by gas chromatography‐mass spectrometry (GC‐MS) and proton transfer reaction‐mass spectrometry (PTR‐MS) [13, 44, 186]. GC‐MS enables reliable compound identification but requires prior collection, limiting its ability to capture real‐time emission dynamics. In contrast, PTR‐MS provides rapid and continuous monitoring of volatile emissions but generally lacks detailed molecular identification. Therefore, developing strategies that combine the complementary advantages of these two techniques remains an important research direction.

Future studies require integrated platforms capable of exploring root volatile‐mediated interactions among diverse organisms, while enabling effective in situ volatile sampling and dynamic chemical analysis. Such systems should also allow the separation of root volatiles from nonvolatile root exudates and facilitate subsequent validation of their ecological effects.

### 7.3 Exploring Ecological Networks

The significance of root volatiles in mediating belowground ecological networks under diverse environmental conditions has not yet been fully characterized. This knowledge gap is mainly due to insufficient understanding of root volatile biosynthesis, release, and diffusion processes, as well as limitations in existing experimental systems, as described above. To further understand these ecological networks, future studies should integrate more diverse biological communities and environmental factors while addressing the following key questions:

1. Relative Contributions of Root Volatiles and Nonvolatile Root Exudates: Root exudates and root volatiles are released simultaneously and jointly influence belowground interactions [39, 102]. However, differences in their persistence and diffusion capacities may determine their distinct ecological functions [103]. Comparative analyses between these two chemical communication pathways will help determine when, where, and toward which organisms root volatiles exert ecological effects.
2. Perception Mechanisms of Root Volatiles by Soil Biota: Existing studies provide several important clues. Insects typically perceive environmental volatile signals through odorant receptors, ionotropic receptors, and gustatory receptors [187, 188], and the involvement of odorant receptors in soil environments has been demonstrated [189]. Microbes may interact with volatile molecules through specific target proteins [113]. Although plants are proposed to perceive exogenous volatiles through multiple mechanisms, such as protein receptors and membrane dissolution [76], direct evidence supporting these perception mechanisms in roots is limited.
3. The Boundary Between Microbial‐ and Root‐Derived Volatiles: An important question is whether the volatile compounds detected in the rhizosphere can be unequivocally attributed to root tissues. Roots harbor diverse endophytic microbes whose volatiles have been demonstrated to play important roles in antifungal defense [190]. Given that these microbes occupy the same place as roots and may contribute to overlapping volatile profiles, whether their ecological functions can be clearly distinguished from those of root‐derived volatiles is an open question.

### 7.4 Applying to Agricultural Systems

Root volatiles have considerable potential for applications in pest management, agricultural practice optimization, and genetic engineering‐based crop improvement [26, 27, 111]. However, their practical application is constrained by several unresolved issues. A limited understanding of volatile biosynthesis restricts the efficiency and precision of targeted breeding strategies. Insufficient knowledge of volatile transport and persistence in soil reduces the reliability of applications across different soil environments. Furthermore, an incomplete understanding of root volatile‐mediated ecological networks limits the establishment of comprehensive ecological risk assessment frameworks. Therefore, an effective application framework should integrate feasible breeding strategies, reliable field performance, and rigorous ecological risk assessment.

## Author Contributions

The authors confirm their individual contributions to the work as follows: XS: conceptualization. XS and XC: methodology (scope and structure design). XC and NS: investigation (literature search). XS, XC, NS, FV, and MP‐H: writing – original draft preparation. All authors: writing – review & editing (commenting on versions). HG, ACM, and AC: writing – review & editing (critical revision for intellectual content). All authors have made substantial contributions to this work.

## Funding

This work was supported by the following grants: the National Key Research and Development Program of China (2023YFE0122100), the National Natural Science Foundation of China (32371752), and the Natural Science Foundation of Henan (232300421027) to XS; the Ramón y Cajal grant from the Spanish Ministry of Science and Innovation (RYC2022‐036061‐I) to MP‐H; the China–France CAAS‐INRAE Associated International Laboratory in Plant Protection (BIPi) to EJ‐J; and the National Natural Science Foundation of China (32372627) to HG.

## Conflicts of Interest

The authors declare no conflicts of interest.
